# Examining the Effects of Quercetin on Phenotypic Characteristics of Human Mesenchymal Stem Cells

**DOI:** 10.1007/s12195-025-00849-y

**Published:** 2025-05-30

**Authors:** Thomas Needy, David Heinrichs, Vitali Maldonado, Ryan Michael Porter, Hanna Jensen, C. Lowry Barnes, Rebekah Margaret Samsonraj

**Affiliations:** 1 Department of Biomedical Engineering, University of Arkansas, 700 W Research Center Boulevard, Fayetteville, AR USA; 2 Department of Orthopedics, University of Arkansas for Medical Sciences, Little Rock, AR USA; 3 Department of Surgery, University of Arkansas for Medical Sciences Northwest Regional Campus, Fayetteville, AR USA

**Keywords:** Mesenchymal stem cells, Senescence, Senolytics, Quercetin, Cell Therapy

## Abstract

**Introduction:**

A significant obstacle to mesenchymal stem cell (MSC) potency and therapeutic utility is in vitro senescence, an irreversible cessation of replication associated with age-related complications. Senolytic drugs, such as quercetin, may be helpful in selectively culling senescent cells while leaving non-senescent cells unaffected, thereby increasing potency of high-passage MSCs.

**Methods:**

The phenotypic, genotypic, and immunomodulatory effects of quercetin were assessed using in vitro models. Senescent cells, created through repeated subculturing of MSCs in vitro, and non-senescent cells were treated with 10 μM quercetin, differentiated into osteocytes, adipocytes, and chondrocytes, and analyzed to observe the effect of quercetin.

**Results:**

Quercetin was not found to be beneficial to MSC function. It did not exhibit a consistent senolytic effect as evidenced by SAβ-gal and live dead staining, hindered proliferation in the short term in some donors, and lowered the expression of osteogenic markers COL1A1 and ALP. Quercetin treatment did not, however, negatively affect adipogenesis, chondrogenesis, or indoleamine 2,3 dioxygenase secretions.

**Conclusion:**

This study contributes insight into the nature of quercetin and its effects on in vitro MSC culture and function.

## Introduction

Mesenchymal stem cells (MSCs) are characterized by their ability to self-renew, form colonies, and differentiate into osteoblasts, chondrocytes, and adipocytes [1]. MSCs can be isolated from bone marrow, adipose tissue, umbilical cord, endometrial polyps, and menstrual blood, among other sources. MSCs are a type of stromal cells and present an advantage in research over other types of stem cells, such as embryonic stem cells, because MSCs possess a relatively low rate of tumor formation and lack ethical controversy. MSCs can be identified by their lack of CD34, CD45, CD14, HLA-DR, and CD11b proteins and their presence of CD105, CD73, and CD90 proteins on the cell surface. MSCs may be isolated from culture due to their plastic-adhering properties [2]. MSCs are ideal candidates for musculoskeletal repair and regeneration therapy due to their secretion of growth factors, cytokines, interleukins, and chemokines. MSC immunoregulatory pathways involve cell-to-cell contact and paracrine signaling, secreting factors that can help suppress local immune responses, thus helping other local stem cells promote tissue healing. Evidence has also shown that MSCs may be beneficial in treating various orthopedic regenerative diseases. The specific type of MSC used in this study was bone marrow-derived MSCs (BM-MSCs). BM-MSCs are the most widely utilized MSC owing to their relative ease of isolation and culture, abundance, and robust differentiation capability [3, 4].

For MSCs to be effective in stem cell therapy, they must be potent [1]. MSC potency is a measure of the ability to perform regenerative and immunomodulatory functions that can be assessed both in vitro and in vivo. In vitro, potency can be measured by evaluating the MSC’s differentiation ability and ability to secrete various growth factors and bioactive molecules. Potency is an effective predictor of the efficacy of an MSC culture and, therefore, its therapeutic potential [5]. The primary factor affecting MSC potency we have investigated in this study is replicative aging, or senescence.

Cellular senescence is an irreversible replicative halt within the cell cycle that causes resistance to apoptosis and increased metabolic activity. Senescence is brought on by cellular stressors such as telomere shortening, DNA damage, oxidative stress, and oncogene activation [6]. Senescence occurs under physiological conditions to protect against cancer and to aid in tissue formation when localized scarring and inflammation are needed. In normal and healthy cells, senescence is intended to be temporary. Once senescent cells have fulfilled their biological functions, the immune system removes them through apoptosis. The detrimental effects of senescent cells arise when they persist in tissues for prolonged periods, failing to be efficiently cleared by immune surveillance mechanisms [7]. Senescence causes alterations in cellular gene expression and epigenetic changes to DNA, and through the Senescent Associated Secretory Phenotype (SASP) can cause neighboring cells to become senescent. The SASP can compromise neighboring cells through the secretion of pro-inflammatory cytokines and growth factors such as TNF-α, IL-1β, SDF-1, and MMP-13 [6–9]. Multiple publications have shown that the accumulation of senescent cells is associated with aging and numerous diseases [8]. Senescent cell accumulation has also been shown to be detrimental, as it reduces cellular health span, the period where cells function in a healthy and optimal state [7]. MSCs extensively cultured in labs can become senescent after multiple rounds of replication as the telomeres shorten. One method of clearing senescent cells is through treatment with senolytic drugs.

Senolytics are a type of drug that selectively clears senescent cells and leaves normal cells intact. Some common senolytics include quercetin, dasatanib, fisetin, and navitoclax [8]. Senolytics function by inhibiting the senescent cells’ apoptosis prevention pathways. Both murine and in vitro models have shown that treatment with senolytic drugs can delay or prevent frailty, cancers, diseases, and complications from treatments such as radiation and chemotherapy [8]. Senolytic drug treatment has also been shown to cause improvements in tissue function and to increase cellular lifespan. One study has shown that elderly and osteoporotic mice treated with senolytics have increased bone density and volume [6], while another investigating mice treated long-term with senolytics showed an increased life span without an increase in cancer or signs of impaired tissue repair [7]. Treatment with senolytics is particularly effective because it does not prevent senescent cells from executing their purpose—it prevents them from lingering and bringing about negative consequences.

The senolytic investigated in this study is quercetin, an antioxidant flavonoid notably common in the Mediterranean diet and found in certain fruits and vegetables [10]. While quercetin used in combination with dasatanib has often shown positive effects on the function of diseased tissue [9, 11, 12], this study was interested in individually examining quercetin’s impacts, especially in the contexts of in vitro MSC culture. This was done so that the individual effects of quercetin could be analyzed independently of dasatanib, thus removing any possible confounding effects. Oral consumption of quercetin in the diet has been related to a reduced risk of cancer and cardiovascular disease, suggesting that it could potentially be a nutraceutical [13]. According to clinicaltrials.gov, as of April 17th, 2025, there are six studies investigating quercetin in cancer settings, four in cardiovascular settings, and thirty-nine in various other settings within the United States. Though the nature of how quercetin works is still not completely understood, studies show that it can exert a wide array of biological effects owing to its anti-inflammatory and antioxidant properties. Namely, literature has shown that quercetin may inhibit inflammatory pathways such as cyclooxygenase-2, nuclear factor-kappa B, activator protein 1, mitogen-activated protein kinase, reactive nitric oxide synthase, and reactive C-protein [14].

Quercetin and its effects against malignant age-induced phenotypes have been investigated on numerous models, including rodents, non-human primates, non-human MSCs, and human MSCs. Its effects as a senolytic agent have been mixed, with some studies demonstrating significant positive effects, others demonstrating significant negative effects, and others showing no effect. In contrast to other studies, this one offers a more multifaceted and comprehensive analysis, using both senescent and non-senescent cells to first assess quercetin’s senolytic efficacy and then analyze its holistic impacts on in vitro MSC culture through growth, differentiation, gene expression, and immunomodulation assays. The results of this study indicate donor-dependent variability and inconsistent impacts following quercetin treatment, challenging optimism in its therapeutic efficacy as a monotherapy.

## Materials and Methods

### Culture of MSCs

Bone marrow-derived MSCs from male donors between 20 and 30 years old were bought from Lonza and cultured in a maintenance media. The maintenance media was Dulbecco’s Modified Eagle’s medium (DMEM) and consisted of 1 g/1 glucose, 10% fetal calf serum, 2 mM 1-glutamine, and 50 U/mL streptomycin. The cells were frequently passaged at 80% confluency using ThermoFisher TrypLE Express (Cat. No. 12605010). The media was changed every 3–4 days, and the cells were kept in a humidified incubator at 37 °C and 5% CO_2_ to ensure the cells had optimal environmental conditions. Low-passage, low-senescent and high-passage, high-senescent cells from 3 individual donors were used for all experiments. When the non-senescent cells were plated, they were seeded at a density of 3000 cells/cm^2^, while when the senescent cells were plated, they were seeded at a density of 5000 cells/cm^2^. This was done to ensure that cells reached 80% confluency at approximately the same time. The low-passage, low-senescent cells were passaged 5–9 times. Senescent MSCs were created through repeated rounds of subculture and replication. Cells from each donor were divided into four different treatment groups: low-passage cells treated with 10 µM quercetin, low-passage cells treated with dimethyl-sulfoxide (DMSO) as a control, high-passage cells treated with 10 µM quercetin, and high-passage cells treated with DMSO.

### Quercetin Dosage Optimization

The safe ranges of quercetin dosage were determined using the ThermoFisher CyQUANT MTT Cell Viability Assay Kit (Cat. No. V-13154) following the manufacturer’s protocol. Low-passage MSCs were seeded into a 96-well plate at a density of 10,000 cells per well in 100 µL of DMEM. The cells were incubated for 72 h along with quercetin at various concentrations: 500 µM, 250 µM, 125 µM, 62.5 µM, 31.25 µM, 15.625 µM, 7.813 µM, and 0 µM (control). After 72 h of incubation, the media was replaced, 10 µL of MTT stock solution was added to each well, and the cells were incubated for an additional 4 h. Following this, 100 µL of SDS-HCl solution was added to each well, and the cells were incubated for another 4 h. The samples were then mixed thoroughly, and absorbances for each well were measured at 570 nm using a plate reader.

### Live Dead Staining with Nuclear Counterstain

Live/dead staining was conducted using the ThermoFisher Scientific LIVE/DEAD viability/cytotoxicity assay kit (L32250) protocol. High-passage cells from 3 donors were seeded in 35 mm plates at a density of 5000 cells/cm^2^ and allowed to reach approximately 80% confluency. Cells were then treated with quercetin for 3 days and then allowed to recover for 3 days. After the recovery, the cells were washed with PBS and stained with red and green dyes diluted in a working reagent. The cells were incubated for 30 minutes, with DAPI added in the final 15 minutes to stain the nuclei, and were imaged with a fluorescent microscope. Cells were counted using previously optimized, preset conditions on ImageJ.

### Growth Rate Analysis

Low-passage MSCs were seeded into 12-well plates at a density of 3,000 cells/cm^2^ in maintenance media and allowed to reach approximately 60% confluency. Cells were then treated with maintenance media with added quercetin or DMSO as specified by the experimental group for 3 days. After 3 days, the media was changed to maintenance media without additional quercetin or DMSO. Following the treatment period, one row of cells from the 12-well plate was detached, transferred to a 96-well plate, and treated with 10 µL of the Cell counting Kit-8 assay (Tocris Cat. 7368). After 4 h of treatment, absorbance was measured at 450 nm using a plate reader. For donor 172, this process was repeated every two days for 12 days. For donor 257, which exhibited a faster growth rate, measurements were taken every two days for 8 days, with a final measurement on the ninth day. Absorbance values were used to determine the growth rate.

### Senescence β-galactosidase Staining

Senescence β-galactosidase (SAβ-gal) staining was conducted using the protocol from the Cell Signaling Technology kit (Catalog #9860). High-passage MSCs from 3 donors were seeded into 12-well plates at a density of 5000 cells/cm^2^ and allowed to reach approximately 80% confluency. The cells were divided into two groups: the experimental group, treated with quercetin, and the control group, treated with DMSO. Cells were treated for 3 days and given 3 days to recover by changing the media to maintenance media. At the end of the recovery, the cells were washed with PBS and treated with the SAβ-gal kit following manufacturer instructions. Following overnight incubation, the wells were imaged using a microscope at 40x magnification, and senescent cells were counted using previously optimized, preset conditions on ImageJ. DNA was harvested following the protocol from the Qiagen QIAamp DNA Mini Kit (Catalog #51306) and quantified using a spectrophotometer. Senescent cell numbers per well were normalized to the DNA concentration per well. Two-tailed t-tests were utilized to verify whether the observed differences between experimental and control groups were statistically significant.

### Osteogenic Differentiation

The 4 MSC groups were seeded into 6-well plates in maintenance medium according to their age-specified densities and cultured until the cells were approximately 80% confluent. Once the cells were confluent, the maintenance medium was changed to an osteogenic medium. The osteogenic differentiation medium consisted of 10 nM dexamethasone, 25 µg/mL ascorbic acid, and 10 mM beta-glycerophosphate. The medium was also treated with quercetin or DMSO, as specified by the experimental group. The cells were treated with quercetin or DMSO for 7 days according to their group. After this 7-day period, cells were no longer treated with quercetin or DMSO. To visualize osteogenic cells, they were cultured for two additional weeks with media changes every 3-4 days, and after 21 total days, the cells were fixed with 4% formaldehyde, stained with Alizarin Red, and imaged using a microscope.

### Adipogenic Differentiation

The 4 MSC experimental groups were seeded into 6-well plates in the maintenance medium according to their age-specified densities. Once reaching 80% confluency, the maintenance medium was changed to adipogenic medium. The adipogenic medium was high glucose DMEM with 1 µM dexamethasone, 10 µM insulin, 100 µM indomethacin, and 11.5 µg/mL 2-isobutyl-1-methylxanthine. The medium was treated with quercetin or DMSO according to the experimental group, and cells were exposed to this media for 7 days. After the 7-day period, the cells were no longer treated with quercetin or DMSO but continued to be treated with adipogenic media for an additional 21 days. After the 28-day period, the cells were fixed with 4% formaldehyde and stained with Oil-Red-O. The resulting lipid droplets were imaged with a microscope and quantified using ImageJ.

### Gene Expression Analysis

MSCs were seeded in 6-well plates at their age-specified density in the maintenance medium until the cells were approximately 80% confluent. Once cells became confluent, the maintenance media was changed for either control (DMSO) or experimental (quercetin) adipogenic or osteogenic media, depending on the group. Cells were treated for 7 days with media changes every 3–4 days. After 7 days of treatment, the media was replaced with untreated adipogenic or osteogenic media, and cells were treated for another 7 days with media changes every 3–4 days. On the 14th day, the media was removed from the wells, and the cells were lysed using RLT lysis buffer, and the cell lysate was collected. RNA was isolated from the cell lysate using the Qiagen RNeasy kit (Cat. 74104) and diluted to standardize the concentration. Next, the RNA was reverse transcribed into cDNA using a thermocycler and the VILO Superscript cDNA synthesis kit (Invitrogen Ref. 11754-250). Gene-specific primers were added to the cDNA, and real-time quantitative PCR (RT-qPCR) was performed using a thermocycler with SYBR green as the indicator. The osteogenic markers examined were ALP, SP7, and COL1A1. The adipogenic markers analyzed were FABP4 and PPARγ. The data was analyzed using the comparative Ct method, and relative expression units were measured using RPLP0 as the control gene. Differences between control and experimental genes were assessed using t-tests.

### Chondrogenic Differentiation

The MSCs were seeded into round-bottom, non-adherent 96-well plates at a density of 250,000 cells per well in maintenance media as specified by chondrogenesis protocol. After seeding, the plate was centrifuged at 200 rcf for 10 minutes and incubated for 24 h to allow pellet formation. The cells were divided into three groups, each containing three replicates. The control group was treated with only chondrogenic differentiation media (Gibco Ref. A10069-01), the second group was treated with chondrogenic media and transforming growth factor beta (TGF-β) (10 ng/mL), and the experimental group was treated with chondrogenic media, TGF-β, and 10 µM quercetin. MSCs underwent treatment for 7 days with media changes every 3-4 days. After the treatment period, the cells were treated with chondrogenic media with or without TGF-β as specified by the experimental group for an additional 21 days. After the 28-day culture period, pellets were fixed with 4% formaldehyde and embedded in paraffin. Five-micron sections were stained with 0.05% Toluidine Blue Solution in McIlvaine’s Buffer (pH 4.0) [Bergholt NL, Lysdahl H, Lind M, Foldager CB. A Standardized Method of Applying Toluidine Blue Metachromatic Staining for Assessment of Chondrogenesis. Cartilage 2019 Jul; 10(3): 370–374. PubMed ID: 29582671]. Serial sections underwent antigen retrieval at 65 °C for 2 h in sodium citrate buffer (pH 6.0), blocking in 5% goat serum, overnight staining with a rabbit monoclonal anti-SOX9 antibody (ab185966, 1:1000 dilution), and detection using a Vectastain Elite ABC-HRP rabbit kit (PK-6101), with nuclear counterstaining using 0.5% Methyl Green.

### Indoleamine Di-oxygenase (IDO) Activity Assay

MSCs were seeded in a 12-well plate in maintenance media at a density of 3,000 cells/cm^2^ and allowed to reach approximately 80% confluency. Low-passage and high-passage cells were split into four groups: cells exposed to 50 ng/mL interferon-gamma (IFN-γ) and quercetin, cells exposed to IFN-γ and DMSO, cells exposed to quercetin and 10% Fetal Bovine Serum (FBS) in PBS solution (to act as a control to IFN- γ), and cells exposed to FBS solution and DMSO. These groups underwent treatment for 3 days. After 3 days, the cells were lysed using a RIPA buffer and were centrifuged at 10,000 rpm at 4 °C for 10 min to get supernatant debris-free samples for the IDO assay. The IDO assay was performed using the IDO ELISA kit (MyBioSource MBS028821). Data was collected from both the cell lysate and the supernatant, and then the lysate and supernatant were combined to see the total concentration of each experimental group. A one-way ANOVA was performed to find significant differences between the eight groups.

## Results

### Quercetin Dose Optimization

**Fig. 1:**
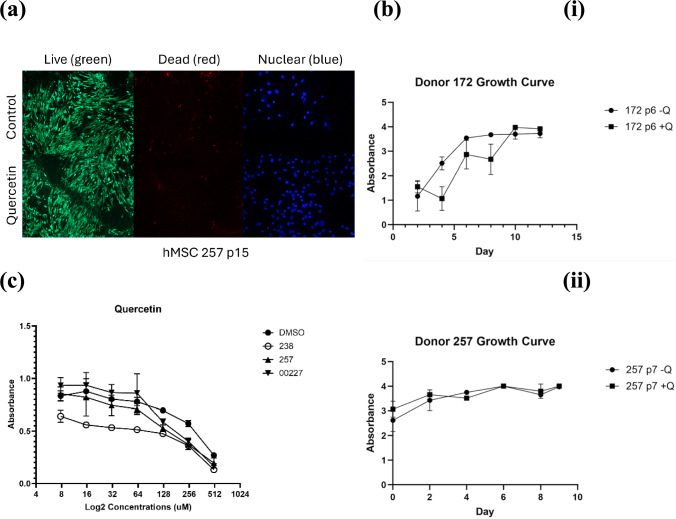
**a** Live/dead stain images of MSCs treated with 10 μM quercetin or DMSO. Living cells are fluorescent green, dead cells are fluorescent red, and cell nuclei are fluorescent blue. **b** Growth curve analyses following 3-day treatment with 10 μM quercetin or DMSO. **i** Growth curve of donor 172. **ii** Growth Curve of donor 257. **c** Dose-response curves to quercetin at varying concentrations

The optimal quercetin dosage was analyzed using a dose-response curve (Fig. 1c), which examined cell viability following quercetin treatment at various concentrations. A safe dose should have an inhibitory effect on cell proliferation yet should not significantly damage the cell culture. The data indicates that 10–90 µM dosages are safe and effective. Based on these results and literature suggesting optimal quercetin dosages are less than 10 µM [15, 16], all further treatments in this study utilized 10 µM quercetin.

### Quercetin has No Clear Impact on Clearance of Senescent Cells

After conducting live dead staining on MSCs from donor 257, it was not clear that quercetin had a significant clearance effect on senescent cells. Qualitative image analysis using ImageJ shows there were 748 living cells per field of view in the control group, while after treatment, the cell count decreased to 674. This equates to a 9.98% decrease in live cell number following treatment and recovery time. Before treatment, there were 43 dead cells, and after treatment, there were 11, resulting in a 74.4% reduction in the number of dead cells. This data (Fig. 1a) does not suggest a widespread clearance carried out by quercetin.

CCK-8 assays were conducted following treatment with quercetin or normalization with DMSO to assess quercetin’s effects on MSC growth rate (Fig. 1b). The data indicates donor-dependent effects of quercetin, as seen by depressed growth following quercetin treatment in donor 172 (Fig. 1bi), and unaffected growth following treatment in donor 257 (Fig. 1bii).

**Fig. 2:**
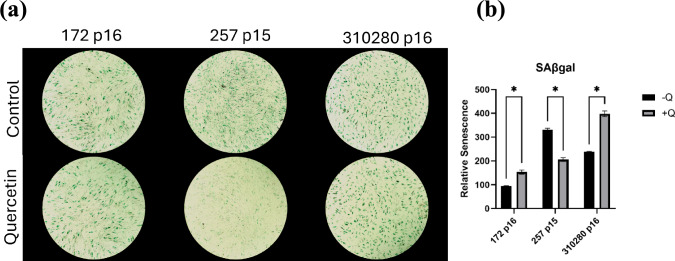
**a** SAβ-gal-stained cells imaged at 40x magnification following quercetin or DMSO treatment. **b** Relative senescence of cells following quercetin or DMSO treatment. Significance (two-tailed *t*-test): *P < 0.05. None detected (ND)

Senescent cell staining shows little consistent qualitative effects of quercetin. While there was some senolytic clearance effect in donor 257, donors 172 and 310280 did not show any qualitatively substantial differences in the number of senescent cells in groups treated or not treated with quercetin (Fig. 2a). When the number of senescent cells was normalized to the concentration of DNA in the respective well, it is noted that quercetin only exhibited a senolytic effect with donor 257 (Fig. 2b).

### Quercetin has No Clear Impact on the Osteogenic Differentiation of MSCs

**Fig. 3:**
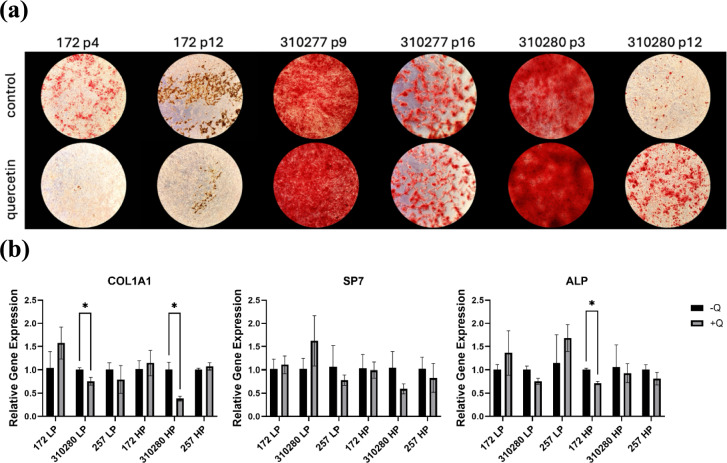
**a** Alizarin Red-Stained osteogenic cultures following 21 days of osteogenesis imaged at 4x magnification. **b** RT-qPCR analysis of osteogenic markers COL1A1, SP7, and ALP following quercetin or DMSO treatment. LP: low passage. HP: high passage. Significance (two-tailed *t*-test): *P < 0.05

MSCs were differentiated into osteoblasts to examine the effects of quercetin on the osteogenic differentiation capacities and relative gene expression levels of low-passage and high-passage cells. Qualitatively, the effects of quercetin were inconsistent and donor-dependent (Fig 3a). At particular stages in culture, control groups exhibited greater mineralization (172 p4 and p12). Conversely, in some donors (310280 p3 and p12), quercetin-treated groups displayed noticeably greater mineralization when compared to controls. In others (310277 p9 and p16), differentiation levels were similar regardless of quercetin’s presence, suggesting pronounced donor-dependent heterogeneity.

RT-qPCR was conducted to analyze the effects of quercetin on osteocytes via the relative levels of gene expression for genes COL1A1, SP7, and ALP (Fig 3b). Quercetin-treated MSCs showed repression of COL1A1 in low-passage and high-passage cells from donor 310280. It also adversely affected the ALP gene expression in high-passage cells from donor 172. In all other groups, the observed differences between donors were within the margin of error and showed inconsistent effects.

### Quercetin has No Clear Impact on the Adipogenic Differentiation of MSCs

**Fig. 4:**
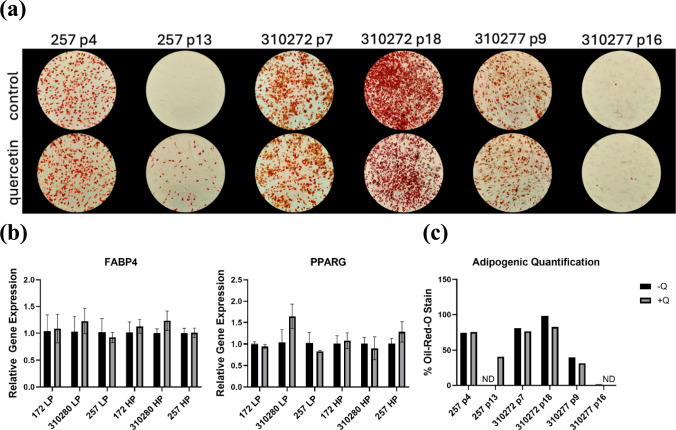
**a** Oil-Red-O stained cultures imaged at 10× magnification following 28 days of adipogenesis. **b** RT-qPCR analysis of adipogenic markers PPARγ, and FABP4 following quercetin or DMSO treatment. LP: low passage. HP: high passage. Significance (two-tailed *t*-test): *P < 0.05. **c** Quantification of imaged adipocytes based on percentage of stained cells relative to total number of cells

In order to determine the effects of quercetin on adipogenic differentiation and levels of relative gene expression in low-passage and high-passage cells, MSCs were differentiated into adipocytes. Following oil-red-O staining and qualitative analysis, quercetin did not appear to have significant and consistent effects on differentiation (Fig 4a). For many donors and passages such as 257 p4, 310272 p7, and 310277 p9 and p16, the observed differences between the control and the treatment groups are negligible. On the other hand, donor 257 p13 demonstrated markedly enhanced differentiation following quercetin treatment, while in donor 310272 p18, the control group exhibited better differentiation. Overall, the staining results do not indicate a consistent effect of quercetin treatment. These findings are further supported by the adipogenic quantification results, which suggested that while donor 257 p13 benefitted from quercetin treatment, most other groups had similar percentages of cells stained (Fig 4c).

Adipogenic markers PPARγ and FABP4 were examined quantitatively using RT-qPCR (Fig 4b). As with the qualitative staining results, the PCR data showed no significant difference between the gene expression of treatment and control groups. Though there appears to be a trend of quercetin-treated groups having a lower expression of FABP4, the differences were not statistically significant. With PPARγ expression, there were no consistent effects, even within the margin of error.

### Quercetin has No Clear Impact on the Chondrogenic Differentiation of MSCs

**Fig. 5:**
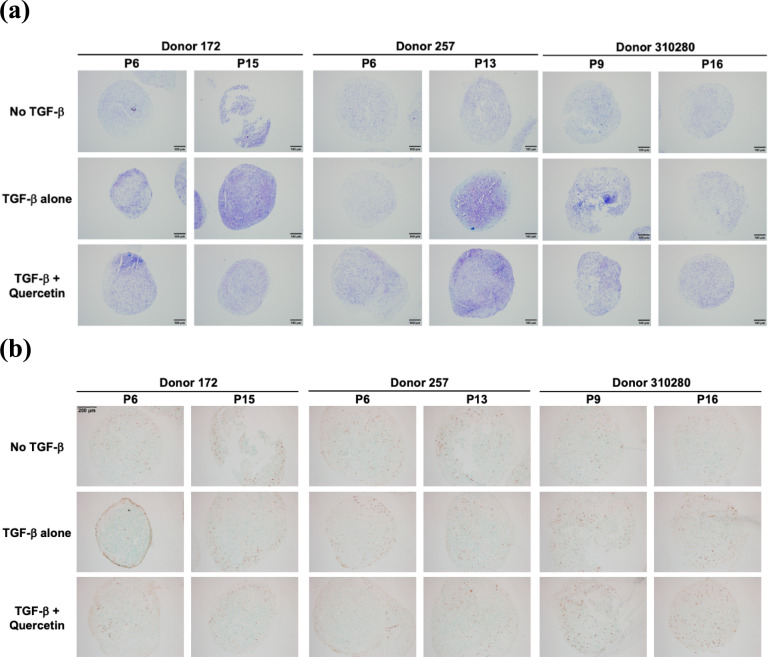
**a** Toluidine blue stained MSC chondrocytes following 28 days of differentiation imaged at 10× magnification. **b** MSC chondrocytes following 28 days of differentiation stained for the SOX9 antibody imaged at 10× magnification

MSCs were differentiated into chondrocytes to determine the effects of quercetin treatment on chondrogenesis (Fig. 5). To visualize chondrocytes, cells were stained using toluidine blue staining (Fig. 5a). This process causes a metachromatic shift from blue to purple in the extracellular matrix (ECM) with binding to sulfated glycosaminoglycans (GAGs). This process is most apparent, resulting in the deepest stain, in the TGF-β treated groups. For further evidence of chondrogenesis, cells were stained for the SOX9 antibody (Fig. 5b). Qualitative analysis of the staining images does not provide conclusive results suggesting quercetin’s effectiveness in promoting chondrogenesis. However, when compared to the TGF-β alone group, adding quercetin does not decrease GAG deposition or SOX9 expression, as seen in the images for cells treated with TGF-β and quercetin.

### Quercetin has No Clear Impact on IDO Activity

**Fig. 6:**
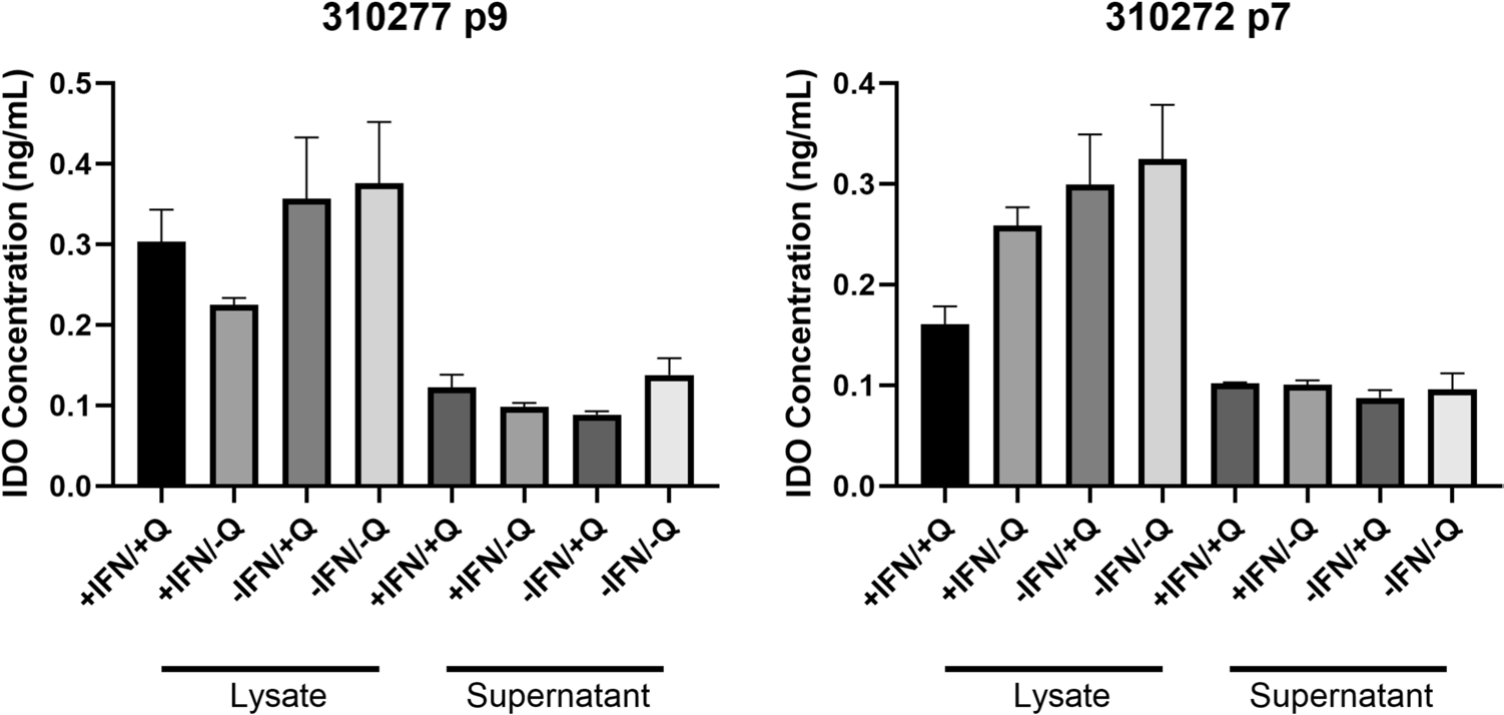
Combined IDO secretion of MSCs treated with quercetin or DMSO and IFNγ. Significance (One-way ANOVA): *P < 0.05

MSCs were analyzed with ELISA for secretion of IDO (indoleamine 2,3-dioxygenase) after short-term treatment with IFNγ and quercetin. IDO activity was highest for (IFNγ-/quercetin-) in both passages assessed. We note that the presence of IFNγ reduces IDO activity much more than the presence of quercetin when analyzing the lysate. However, the effects of quercetin compared to groups untreated with quercetin appear inconsistent (see Fig. 6).

## Discussion

The efficacy of quercetin as a senolytic and its downstream effects remains a topic of notable inconsistency in literature. Reports of optimal dosages vary widely, with some studies describing dosages as low as 10 µM as inhibitory [10], while others observe no senolytic effect even with doses as high as 100 µM [17]. Based on our dose-response curve (Fig. 1c) and studies suggesting that ideal quercetin dosages are less than 10 µM [15, 16], we chose to treat cells with 10 µM quercetin. Overall, our data suggests that quercetin’s effects differ between donors. However, it should be noted that the inconsistent effects of quercetin and the findings contrary to existing studies presented in this investigation can likely be attributed to differences in dosage, treatment duration, and the specific model used. Our results generally indicate that quercetin neither possesses a senolytic effect nor increases MSC potency. In this section, we discuss these findings in the context of senolytic activity, differentiation potential, and immune modulation and compare our findings with existing literature.

Following three-day quercetin treatment, little to no consistent senolytic activity was observed. Live-dead staining showed almost no widespread clearance of cells in the quercetin group. This is evidenced by the qualitatively and numerically similar amounts of live and dead cells between the experimental and control groups. The lack of senolytic effect was further suggested by the SAβ-gal staining, which only showed a reduction of senescent cells following quercetin treatment in donor 257. These findings are consistent with another study which found that despite utilizing high quercetin concentrations (100 µM), no senolytic effect in long-term culture, telomere length rejuvenation, or evidence that quercetin significantly altered the senescence signature was found [17]. In contrast, our findings disagreed with other studies that found significant reductions of senescent cells and other senescent markers, such as reactive oxygen species and inflammatory cytokines, following quercetin treatment [18, 19]. However, it should be noted that both studies suggesting quercetin’s success as a senolytic investigated murine cells, which could explain the differences.

The evidence regarding the proliferative effects of quercetin remains inconsistent across studies, with findings varying significantly depending on factors such as experimental conditions, cell types, dosages, and methodologies employed. Our data suggests that quercetin’s effect is largely donor-dependent. In the growth assay conducted on donor 172, quercetin shows adverse short-term effects on MSC growth rates, but in the long term, it does not affect the maximum number of cells in the culture. This can be seen in Fig. 1bi by the fact that cells in the control group reached confluency at day five and stopped growing for the remainder of the assay, while it took quercetin-treated cells five additional days to catch up and reach confluency. It should be noted, however, that the confluent cells were not sub-cultured, and the limited space was likely the reason for the stall in growth. Conversely, in donor 257, cells treated with quercetin demonstrated no suppression in growth and, at times, outpaced the growth of cells from the control group (Fig. 2bii). The inconsistent reporting of other studies validated our findings: some showed that after 4 days of proliferation, cells treated with quercetin also demonstrated noticeably lower MSC populations than control groups [16], while others found that quercetin significantly enhanced MSC proliferation but notably found that the optimal concentration was lower than the 10 µM we used [15, 20]. This may suggest that due to the higher concentrations we used, there was more of a cytotoxic effect, explaining the depressed growth rate.

In order to analyze the effects of quercetin on osteogenesis, MSCs were differentiated into osteocytes, stained with Alizarin red, imaged, and osteogenic markers were analyzed using RT-qPCR. The three osteogenic marker genes analyzed were ALP, COL1A1, and SP7. ALP is richly expressed in osteoblasts and aids bone development [21, 22]. COL1A1 is a gene essential in type I collagen synthesis, a vital material in osteogenesis [23]. Finally, SP7 is a crucial transcription factor in osteoblast differentiation and bone mineralization that plays a role in intramembranous bone formation and terminal cartilage differentiation [24]. Because of the instrumental roles these genes play in osteocyte formation, it is crucial that quercetin not decrease their relative expression. Although qualitatively, quercetin did not have consistent and significant effects on osteogenic differentiation, gene expression data showed that it either maintained or lowered the expression of the aforementioned markers. Statistically significant reductions in COL1A1 and ALP were observed in some experimental groups, whereas SP7 was not significantly affected. However, the effects appeared to be largely donor-dependent, as not all groups were affected similarly.

It should be noted that while donor 310280 qualitatively demonstrated better mineralization following quercetin treatment, the gene expression results suggested adverse effects of quercetin. This discrepancy can be explained by the fact that RNA was harvested for conversion to cDNA for PCR after 14 days of osteogenesis, whereas imaging was taken after 21 days. This additional week likely gave the quercetin-treated cells ample time to catch up, resulting in the noticeably darker staining**.**

The literature surrounding quercetin’s effects on osteogenesis largely suggests that it is beneficial; however, some studies have found results similar to ours. One study that also investigated a 10 µM dosage of quercetin found that it inhibited ALP activity, mineralization, and downregulated COL1A1 [10]. Meanwhile, many other studies have found that quercetin promotes osteogenic differentiation and promotes ALP expression in addition to other osteogenic markers [15, 16, 20].

Previous studies regarding the effects of senolytics on adipogenesis have proven more promising, suggesting that quercetin modulates, or at least does not hinder adipogenesis. Literature has shown that crucial adipogenic markers such as PPARγ, a master regulator gene essential to adipogenesis [25], and FABP4, a regulator of lipid homeostasis that influences lipid metabolism [26], are enhanced by quercetin treatment [10]. Our findings indicate that quercetin treatment does not significantly help or hinder adipogenesis, as we found little consistent differences between the control and experimental groups. Additionally, our RT-qPCR data showed insignificant differences in PPARγ or FABP4 expression, which is corroborated by other studies [16]. The likely reason for this, as with many other observed differences, can be explained by donor-dependent differences in tolerance for quercetin. Though some investigations suggest that quercetin may modulate adipogenesis, other senolytics, such as dasatanib, appear even more effective, presenting more consistently positive results [27].

From qualitative chondrogenesis analysis, we decipher that the effects of quercetin on MSCs were marginal. While the TGF-β-treated cells and the quercetin and TGF-treated cells demonstrated noticeably higher SOX9 immunohistochemical staining and GAG deposition in the ECM, it is unclear if quercetin contributed any additional benefits to chondrogenesis. It strongly appears not to have any apparent adverse effects. Though there are studies on the impacts of senolytics on chondrogenesis [28], there seems to be a gap regarding quercetin’s specific effects on human MSC chondrogenesis in vitro*.*

Apart from the differentiation capacities, it is vital that quercetin does not interfere with MSC immune function. In line with immune function, MSCs can act to secrete factors such as IL-10, IDO, VEGF, CCL-5 (RANTES), prostaglandin E2, and nitric oxide [1], as well as inhibit B and T cell proliferation [29]. The IDO assay results in this study show no immunocompromising effects caused by quercetin. This finding is strengthened by another study, which shows that quercetin goes even further and enhances IDO secretion [30].

After analyzing the existing body of literature, including this study’s findings, no clear conclusions can be drawn about the efficacy of quercetin. The effects of quercetin treatment appear to be dose and donor-dependent, with notable variability in vitro and in vivo*.* While quercetin, or quercetin and dasatanib treatment appear to be very promising as senolytics in living primate models [31], and murine models [9], its effects in cell culture are convoluted. Perhaps some of the reasons for the successes in in vivo models treated with quercetin and dasatanib are owed more to dasatanib.

## Conclusion

The effects of quercetin on MSC function found in this study range from negligible to sub-optimal. Our findings neither agree nor disagree with existing literature, as many studies report contrasting findings on the effects of quercetin. Our data indicates that quercetin does not consistently eliminate senescent cells, which could be attributed to donor-to-donor heterogeneity. Additionally, it does not seem beneficial to MSC differentiation. Although adipogenesis and chondrogenesis are not significantly impacted by quercetin treatment, gene expression examination shows that quercetin may negatively affect osteogenesis. Despite these findings, IDO levels show that quercetin maintains the immunomodulatory capacity of MSCs. Quercetin treatment alone does not seem to be an effective way to increase the health span of MSCs expanded in in vitro culture. However, different senolytics, such as dasatanib, show signs of being effective. More research needs to be done into other senolytics, particularly investigating long-term studies to examine cellular responses to senolytic treatment over generations.

## Data Availability

Available upon request to corresponding author.
